# Exercise Prevents Memory Impairment Induced by Arsenic Exposure in Mice: Implication of Hippocampal BDNF and CREB

**DOI:** 10.1371/journal.pone.0137810

**Published:** 2015-09-14

**Authors:** Bao-Fei Sun, Qing-Qing Wang, Zi-Jiang Yu, Yan Yu, Chao-Lun Xiao, Chao-Sheng Kang, Guo Ge, Yan Linghu, Jun-De Zhu, Yu-Mei Li, Qiang-Ming Li, Shi-Peng Luo, Dang Yang, Lin Li, Wen-Yan Zhang, Guang Tian

**Affiliations:** 1 Department of Anatomy, Guiyang Medical University, Guiyang, 550004, China; 2 Department of Hospital Infection Management, Affiliated Hospital of Guiyang Medical University, Guiyang, 550004, China; University of Lethbridge, CANADA

## Abstract

High concentrations of arsenic, which can be occasionally found in drinking water, have been recognized as a global health problem. Exposure to arsenic can disrupt spatial memory; however, the underlying mechanism remains unclear. In the present study, we tested whether exercise could interfere with the effect of arsenic exposure on the long-term memory (LTM) of object recognition in mice. Arsenic (0, 1, 3, and 10 mg/ kg, i.g.) was administered daily for 12 weeks. We found that arsenic at dosages of 1, 3, and 10 mg/kg decreased body weight and increased the arsenic content in the brain. The object recognition LTM (tested 24 h after training) was disrupted by 3 mg/ kg and 10 mg/ kg, but not 1 mg/ kg arsenic exposure. Swimming exercise also prevented LTM impairment induced by 3 mg/ kg, but not with 10 mg/ kg, of arsenic exposure. The expression of brain-derived neurotrophic factor (BDNF) and phosphorylated cAMP-response element binding protein (pCREB) in the CA1 and dentate gyrus areas (DG) of the dorsal hippocampus were decreased by 3 mg/ kg and 10 mg/ kg, but not by 1 mg/ kg, of arsenic exposure. The decrease in BDNF and pCREB in the CA1 and DG induced by 3 mg/ kg, but not 10 mg/ kg, of arsenic exposure were prevented by swimming exercise. Arsenic exposure did not affect the total CREB expression in the CA1 or DG. Taken together, these results indicated that swimming exercise prevented the impairment of object recognition LTM induced by arsenic exposure, which may be mediated by BDNF and CREB in the dorsal hippocampus.

## Introduction

Arsenic is a naturally occurring metalloid, which is widely distributed in the environment. Arsenic is found in nature commonly in compounds with oxygen, chlorine, and sulfur, which are called inorganic arsenic compounds. In plants and animals, arsenic combines with carbon and hydrogen, which is called organic arsenic. In general, organic arsenic is usually far less poisonous than inorganic arsenic. The incidences of arsenic poisoning usually occurs in occupational environmental exposure. Chronic exposure to arsenic induces arsenic toxicity, including serious injury to internal organs, subclinical nerve injuries, peripheral neuropathy, and diverse effects on the central nervous system [1]. An estimate 100 million people around the world are exposed to high concentrations of arsenic via drinking water, which has been recognized as a global health problem [2].

Recently, it has been demonstrated that exposure to arsenic could induce alterations in memory and attention [3–6]. Long-term exposure to arsenic significantly affected pattern memory and switching attention in students [4]. The arsenic concentration in urine showed an inverse association with the performance in several cognitive tests in children, indicating that arsenic contamination could affect cognitive development [6]. It was further demonstrated that exposure to the inorganic arsenic, sodium arsenite, in pregnant dams and offspring pups impaired their learning and memory functions [7]. In addition, the effect of exposure to sodium arsenite on spatial memory has been measured. Daily exposure to 15 mg/ kg sodium arsenite significantly disrupted the performance in the Morris water maze and Y maze tasks [8]. Exposure to realgar, an arsenic sulfide mineral, for 6 weeks also affected the cognitive ability of weaned rats in the Morris water maze test and object recognition task [9].

The mechanisms underlying memory impairment induced by arsenic exposure have been mainly focused on arsenic-induced alterations in the hippocampus. Arsenic exposure has been shown to decrease the expression of the 2A subunit of N-methyl-D-aspartate receptor (NMDAR) gene in the rat hippocampus [10]. Moreover, the protein expression of NMDAR 2A subunit, postsynaptic density protein 95 (PSD95) and phosphorylated-Ca2+/calmodulin-dependent protein kinase II alpha in the hippocampus was also decreased in rats exposed to arsenic, whereas the protein expression of synaptic GTPase activating protein, a negative regulator of mitogen-activated protein kinases, was increased [11]. The expression of metabotropic glutamate receptor 5 mRNA and protein in the hippocampus were decreased by arsenic exposure, which may underlie the impaired learning and memory ability observed in rats [12]. Moreover, the arsenic metabolites of realgar are permeable through the blood-brain barrier and accumulate in the hippocampus, which induces excessive glutamate in the extracellular space and excitotoxicity. The excessive glutamate subsequently promotes the activities of glutamine synthetase and phosphate-activated glutaminase, inhibits glutamate transporter 1 expression, and alters NMDAR expression [9]. Arsenic can also change the ultrastructure in hippocampal neurons and induce pathological alterations of neurons and endothelial cells in the hippocampus [9,10]. It has been demonstrated that arsenite reduces the expression of brain-derived neurotrophic factor (BDNF), a critical factor involved in learning and memory, in SH-SY5Y cells [13]. However, the role of hippocampal BDNF in arsenic-induced memory impairment remains unknown.

Numerous studies have concluded that exercise promotes memory performance [14–16], although the underlying mechanism still remains unclear. BNDF in the brain plays a critical role in memory performance and cognition [17–19]. It has been shown that exercise increases cell division and promotes BNDF release, tryosine receptor kinase B (TrkB) activation, and synapsin-1 expression in the dentate gyrus (DG) of the hippocampus, which indicate that BDNF-dependent signaling cascades in the hippocampus are critical for exercise-induced improvement in spatial memory [20]. Forced swimming and running exercises similarly promote cognitive- and brain- related tasks, which appear to be mediated by the BDNF pathway in the hippocampus [21]. A recent study showed that voluntary running wheel or forced treadmill exercises improved memory retrieval and prevented diet-induced cognitive decline, which may be mediated by hippocampal BDNF [22]. Thus, we hypothesized that exercise could improve the impaired memory performance induced by arsenic and that hippocampal BDNF might be involved in this effect.

In the present study, we examined the effect of swimming exercise on arsenic-induced memory impairment in mice objective recognition task, which is a widely used memory model. Furthermore, we tested whether hippocampal BDNF was associated with arsenic exposure and alterations in memory performance. Using behavioral paradigm to alleviate arsenic-induced memory impairment would provide a promising treatment for arsenic toxicity.

## Materials and Methods

### Animals

Male Kunming mice, weighing 20–22 g, were obtained from the Laboratory Animal Center, Guiyang Medical University. Mice were ordered two weeks before each experiment, and a total of 208 mice were used in this study. Mice were housed at a temperature of 21 ± 2°C and humidity of 50 ± 5%, which was controlled by the animal facility with free access to food and water. They were maintained on a reverse 12 h/12 h light/dark cycle. Animals were chosen randomly for each group in all experiments. Each home cage contains four or five mice, which were assigned to the same group. No mice were excluded from each experiment.

### Ethics Statement

All of the experimental procedures were performed according to the National Institutes of Health Guide for the Care and Use of Laboratory Animals and were approved by the Biomedical Ethics Committee for animal use and protection of Guiyang Medical University.

### Arsenic exposure protocol

Mice were randomly assigned to groups and intragastrically administered 0.5 ml of tap water, containing 0, 1, 3 and 10 mg/ kg arsenic at 2:00 PM. daily for three months. The high dosage (10 mg/ kg) was 1/5 of a LD_50_ dosage of arsenic. The arsenic-containing tap water was freshly prepared every day immediately prior to administration. The experimenters carefully attended mice that treated with arsenic twice daily to test pain reflex and observe the toxicity of arsenic. In the present study, mice treated with 1, 3 and 10 mg/ kg arsenic for 12 weeks did not show visible tumor or apparent sickness except for weight loss.

After three months of arsenic exposure (intragastric administration of arsenic), the brain samples were collected from 8 mice in each group for the atomic absorption spectrophotometry test to examine the concentrations of arsenic in the brain.

### Object recognition memory

On the day of the experiment, the mice were placed in a new environment 2 h prior to the start of the test. The test was performed at 10:00 AM. Afterwards, the animals were placed in a white plastic box (55 × 35 × 20 cm). A black metal box (4 × 6 cm) and a yellow glass cone (4 × 6 cm) were used as objects to discriminate (object A1 and object A2). The two objects were placed in two opposite corners, with the center of the objects placed 24 cm from the corner. Next, the mice were placed in the middle of the arena and presented with two identical objects (object A1 or object A2) for a 5-min period (counterbalanced design). Twenty-four hours later, the mice were placed in the same plastic box again and presented with two objects (object A1 and object B) for the 5 min recognition session. Object A2 was always the object that was replaced. Mouse sniffing or touching of the object with its nose and/or forepaws was defined as object exploration. The total time spent with each object in each test was recorded. As mice inherently prefer to explore novel object, then a preference for the novel object (more time than chance [10 sec] with the novel object) indicates intact memory for the familiar object. The plastic box and objects were cleaned with 10% ethanol between each mouse and session.

### Swimming exercise

The swimming exercise was adapted from a previous study [23]. The swimming exercise and arsenic exposure began on the same day. Water temperature during the swimming exercise was maintained at 32 ± 2°C. The exercise groups of mice underwent the swimming exercise for 4 hours prior to daily arsenic exposure. The swimming apparatus was a water glass tank (40 × 60 × 55 cm) filled with tap water (35 cm in depth). No mice were escaped from the water tank in all experiments. Water bubbling was produced by tubes in the bottom connected to an air pump system to prevent floating during the swimming session. The exercise groups of mice were forced to swim for 60 min daily for 12 weeks. All groups of mice underwent swimming exercise showed normal swimming ability. The no exercise groups of mice were housed in the home cage without swimming exercise for the same duration.

### Western blot assays

All of the mice were decapitated without anesthesia, and their brains were rapidly extracted and placed on ice. The CA1 and DG areas were freshly harvested and placed in a 1-ml microtube that contained ice-cold RIPA lysis buffer (Beyotime Biotechnology, Haimen, China). After homogenization using a glass homogenizer, the homogenate was centrifuged at 12 000 × *g* for 10 min at 4°C to obtain the supernatant. The protein concentrations of the samples were determined using the BCA assay kit (Beyotime Biotechnology). The samples were then subjected to sodium dodecyl sulfate-polyacrylamide gel electrophoresis for approximately 30 min at 80 V in the stacking gel and approximately 1.5 h at 120 V in the resolving gel. Proteins were then transferred electrophoretically to Immobilon-P transfer membranes (Millipore, Bedford, MA, USA) at 0.25 A for 30 min. The membranes were washed with TBST (Tris-buffered saline plus 0.05% Tween-20, pH 7.4) and subsequently placed in blocking buffer (5% skimmed dry milk in TBST) overnight at 4°C. The membranes were then incubated for 1 h at room temperature with a primary antibody against BDNF (1:1000), pCREB (1:1000), CREB (1: 1000), and β-actin (1:500) in TBST plus 5% bovine serum albumin. All of the primary antibodies were purchased from Santa Cruz Biotechnology, Santa Cruz, CA, USA. The membranes were washed with TBST buffer for 5 min and then incubated for 45 min at room temperature with secondary antibody (1:5000; Santa Cruz Biotechnology, Santa Cruz, CA, USA). The membranes were washed with TBST buffer for 5 min. Subsequently, the membranes were incubated with a mixture of Super Signal enhanced chemiluminescence substrate (Pierce Biotechnology, Rockford, IL, USA) for 1 min at room temperature. Finally, the blots were exposed against X-ray film (Eastman Kodak Company). The band intensities were quantified using Quantity One software (version 4.0.3) from Bio-Rad (Hercules, CA, USA).

### Statistical analysis

The data were expressed as the mean ± SEM. Repeated measures analysis of variance (ANOVA) was performed to analyze the body weight gain. Two-way ANOVA was performed to analyze the object recognition memory. One-way analysis of variance (ANOVA) was performed to analyze the western blotting results. Post hoc analyses of significant effects in the ANOVAs were performed using Bonferroni's multiple comparisons test. P-values < 0.05 were considered statistically significant.

## Results

### Arsenic exposure decreased body weight and increased the arsenic content in the brain

**Fig 1:**
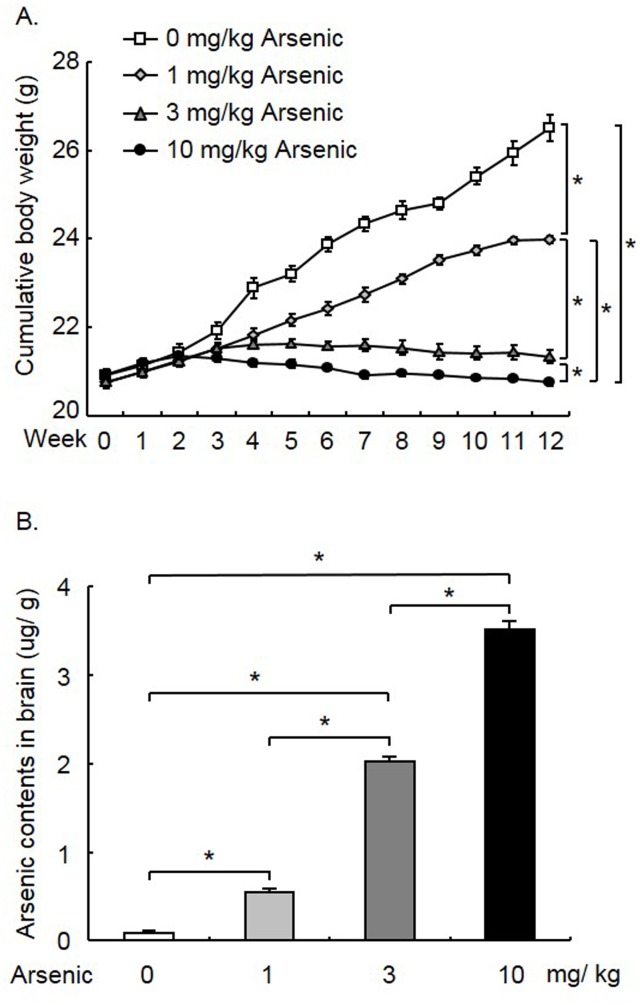
Arsenic exposure decreased body weight gain and increased the arsenic content in the hippocampus. Mice were administered arsenic daily for 12 weeks, and the body weight of mice was recorded weekly. The arsenic content was measured 1 day after arsenic exposure. **(A)** The body weight gain was decreased by 1, 3, and 10 mg/ kg arsenic exposure (*p* < 0.05). The effect on body weight was stronger in mice treated with higher doses of arsenic. **(B)** Chronic arsenic exposure increased the arsenic content in the brains of the mice (*p* < 0.05). Higher arsenic content was associated with a higher dose of arsenic exposure. The data were expressed as the mean ± SEM (n = 12 per group). *p < 0.05, compared between two groups.

During the twelve weeks of arsenic exposure, the body weight of the mice was monitored. Four groups of mice were included in this experiment: 0, 1, 3, and 10 mg/ kg arsenic (n = 12 per group). Repeated measures ANOVA with Group as the between-subjects factor and Time (every week) as the within-subjects factor showed effects of Group (*F*_3, 44_ = 213.88, *p* < 0.001), Time (*F*_12, 44_ = 148.81, *p* < 0.001) and the interaction of Group × Time (*F*_36, 44_ = 58.38, *p* < 0.001). Post hoc analysis showed that chronic exposure to arsenic at a dosage of 1, 3, and 10 mg/ kg significantly inhibited the increase in body weight (compared with 0 mg/ kg arsenic group; all *p* < 0.05). Higher dosages of arsenic induced a stronger effect on body weight gain (all *p* < 0.05; Fig 1A). Taken together, these results indicated that arsenic exposure dose-dependently reduced the increase in body weight.

The arsenic contents in the brains of all mice in this experiment were measured after arsenic exposure. One-way ANOVA showed a significant effect (*F*_3, 47_ = 614.50, *p* < 0.001). Post hoc analysis showed that 12 weeks of arsenic exposure at a dosage of 1, 3, and 10 mg/ kg increased the arsenic content in the brain (compared with 0 mg/ kg arsenic group; all *p* < 0.05). Moreover, the arsenic content in the brain was positively associated with the dosage of arsenic exposed (all *p* < 0.05; Fig 1B). Chronic exposure to arsenic dose-dependently elevated arsenic content in the brain.

### Arsenic exposure disrupted object recognition memory

**Fig 2:**
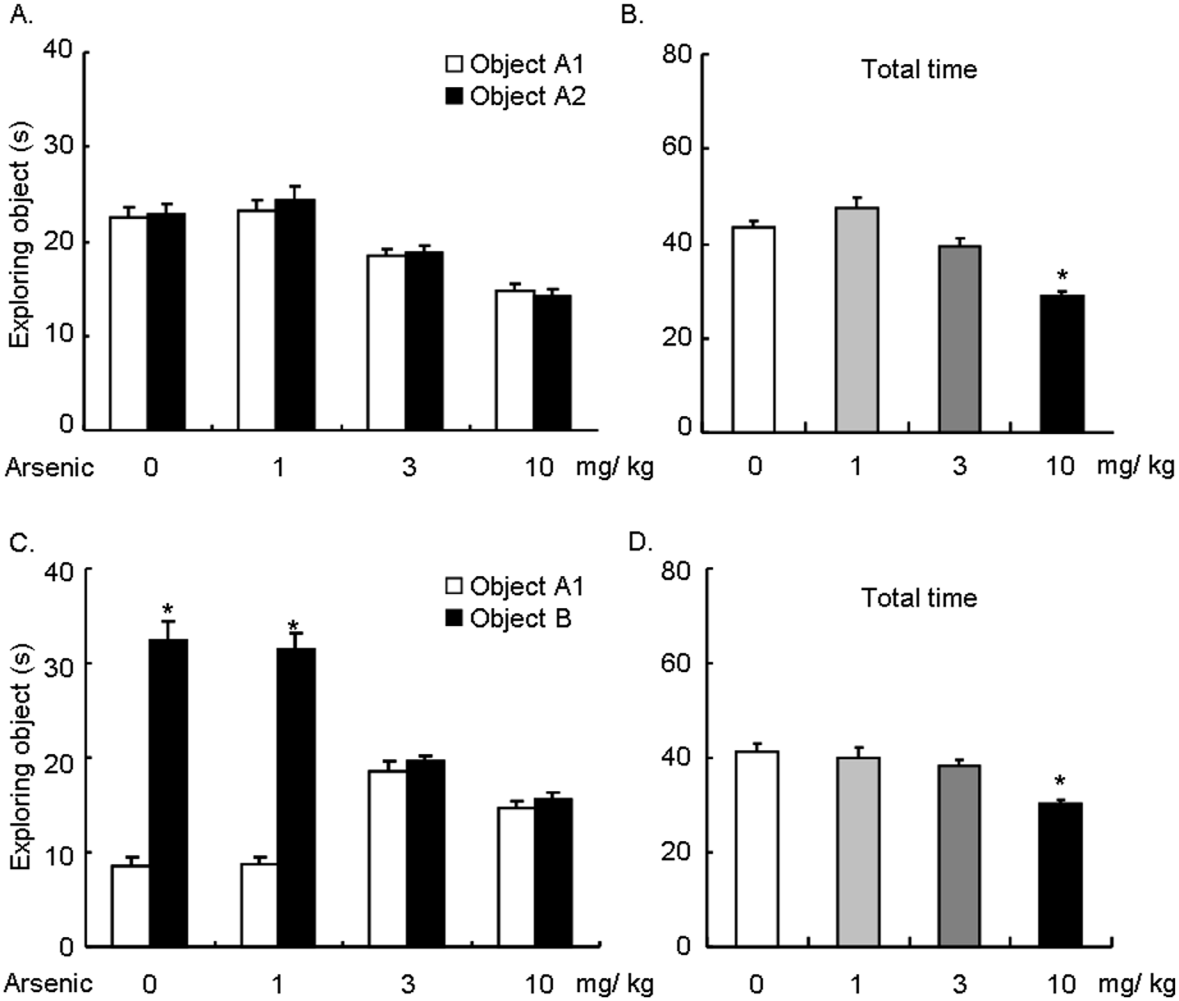
Arsenic exposure disrupted object recognition memory. Mice were administered arsenic daily for 12 weeks and subsequently trained in the object recognition memory task. Long-term memory was tested 24 h later. **(A)** No differences were found in the exploration time in all groups of mice when exploring two identical objects (*p* > 0.05). **(B)** Mice treated with 10 mg/ kg arsenic showed decrease in the total exploring time for both two identical objects (*p* < 0.05). **(C)** When exploring two different objects, groups of mice treated with 0 and 1 mg/ kg arsenic showed preference towards the novel object (both *p* < 0.05). No differences were found in the exploration time in groups of mice treated with 3 and 10 mg/ kg arsenic (both *p* > 0.05), indicating that LTM in these two groups of mice was disrupted. **(D)** Mice treated with 10 mg/ kg arsenic showed decrease in the total exploring time for both two different objects (*p* < 0.05). The data were expressed as the mean ± SEM (n = 14 per group). *p < 0.05, compared with the exploration time to object A1 or 0 mg/ kg group.

Previous studies have demonstrated that arsenic exposure disrupted spatial memory, including the Morris water maze and Y maze test in rats [8,12]. Recently, it was demonstrated that realgar, a type of mineral drug containing arsenic, impaired the object recognition task in rats. Here, we tested whether arsenic exposure could affect object recognition long-term memory in mice. Four groups of mice were included in this experiment: 0, 1, 3, and 10 mg/ kg arsenic (n = 14 per group). One day after 12 weeks of arsenic exposure, the mice underwent memory training, and their memory was tested 24 h later. Two-way ANOVA, with Group as the between-subjects factor and Test object (object A1 and object A2, or object A1 and object B) as the within-subjects factor, was performed to analyze the object recognition LTM. For exploration of the two identical objects, ANOVA showed no significant difference (*F*_3, 52_ = 0.27, *p* > 0.05; Fig 2A). We then analyzed whether arsenic would affect the total exploring time for both objects. One-way ANOVA showed an effect (*F*_3, 52_ = 20.93, *p* < 0.001). Only 10 mg/ kg arsenic significantly reduced the total exploring time (*p* < 0.001), suggesting that 10 mg/ kg arsenic affected locomotion in mice. For exploration of two different objects, ANOVA showed effects in Group (*F*_3, 52_ = 3.08, *p* < 0.001), Test object (*F*_3, 52_ = 220.00, *p* < 0.001), and the interaction of Group × Test object (*F*_3, 52_ = 62.24, *p* < 0.001). Post hoc analysis showed that mice exposed to 0 and 1 mg/ kg arsenic preferred the novel object, which indicated successful LTM (0 mg/ kg arsenic group: *t* = 14.57, *p* < 0.001; 1 mg/ kg arsenic group: *t* = 13.92, *p* < 0.001). Moreover, LTM was impaired in mice exposed to 3 and 10 mg/ kg arsenic (3 mg/ kg arsenic group: *t* = 0.61, *p* > 0.05; 10 mg/ kg arsenic group: *t* = 0.57, *p* > 0.05; Fig 2B). One-way ANOVA analyzing the total exploring time showed an effect (*F*_3, 52_ = 10.08, *p* < 0.001). The 10 mg/ kg arsenic group also showed decrease in the total exploring time (*p* < 0.001). Taken together, these results demonstrated that chronic arsenic exposure disrupted long-term object recognition memory in mice.

### Exercise prevented the impairment of object recognition memory resulting from a low dosage of arsenic exposure

**Fig 3:**
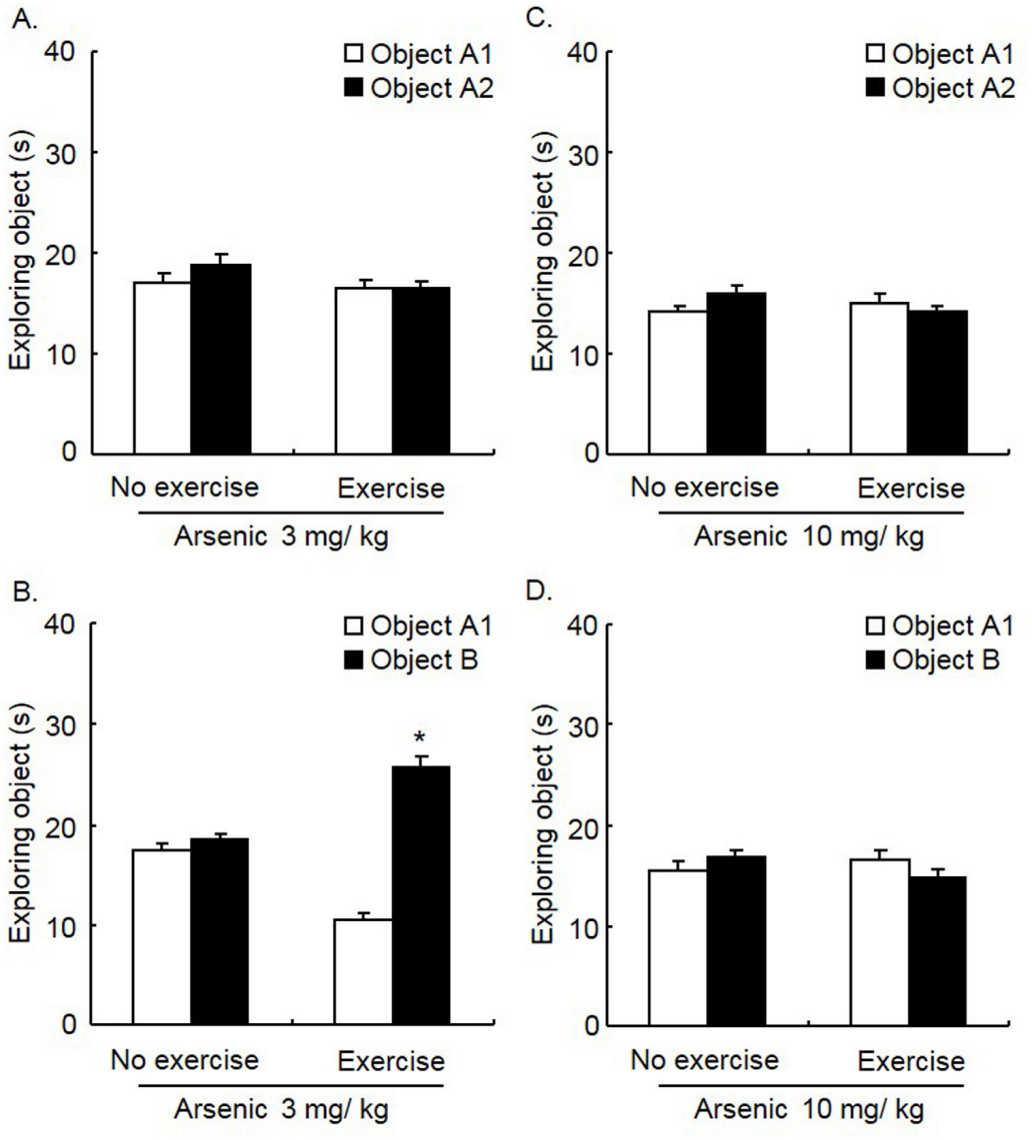
Exercise prevented the impairment of object recognition memory resulting from low doses of arsenic exposure. Mice were administered arsenic and underwent swimming exercise daily for 12 weeks and were subsequently trained in the object recognition memory task. Long-term memory was tested 24 h later. **(A)** Exercise had no effect on the exploration time in mice treated with 3 mg/ kg arsenic when exploring two identical objects (*p* > 0.05). **(B)** Exercise increased the exploration time to novel object in mice treated with 3 mg/ kg arsenic (*p* < 0.05). **(C, D)** Exercise had no effect on the exploration time in mice treated with 10 mg/ kg arsenic when exploring two identical objects or exploring two different objects (both *p* > 0.05). The data were expressed as the mean ± SEM (n = 14 per group). *p < 0.05, compared with exploring time to object A1.

Exercise has been demonstrated to be a promising non-pharmacological approach with neuroprotective effects. In this experiment, we determined whether swimming exercise could affect memory impairment induced by arsenic exposure. The mice were divided into a “no exercise group” (mice without swimming) and “exercise group” (mice with swimming) prior to the experiment. Arsenic was administrated daily for 12 weeks. Two-way ANOVA, with Group (No exercise, and Exercise) as the between-subjects factor and Test object (object A1 and object A2, or object A1 and object B) as the within-subjects factor, was performed to analyze the object recognition LTM. In the experiment using 3 mg/ kg arsenic exposure, two groups of mice were included (n = 14 per group). For exploration of two identical objects, ANOVA showed no effect (*F*_1, 26_ = 1.83, *p* > 0.05; Fig 3A). For exploration of two different objects, ANOVA showed effects of the Test object (*F*_1, 26_ = 85.65, *p* < 0.001) and interaction of Group × Test object (*F*_1, 26_ = 63.20, *p* < 0.001). Post hoc analysis showed that exercise significantly prevented LTM impairment induced by arsenic exposure (*t* = 12.17, *p* < 0.001; Fig 3B). In the experiment for 10 mg/ kg arsenic exposure, two groups of mice were included (n = 14 per group). For exploration of two identical objects, ANOVA showed no effect (*F*_1, 26_ = 4.15, *p* > 0.05; Fig 3C). For exploration of two different objects, ANOVA also showed no significant effect (*F*_1, 26_ = 3.35, *p* > 0.05; Fig 3D). Taken together, these results indicated that exercise prevented memory impairment induced by arsenic at lower dosages.

### Exercise prevented the decrease in hippocampal BDNF induced by low dosages of arsenic exposure

**Fig 4:**
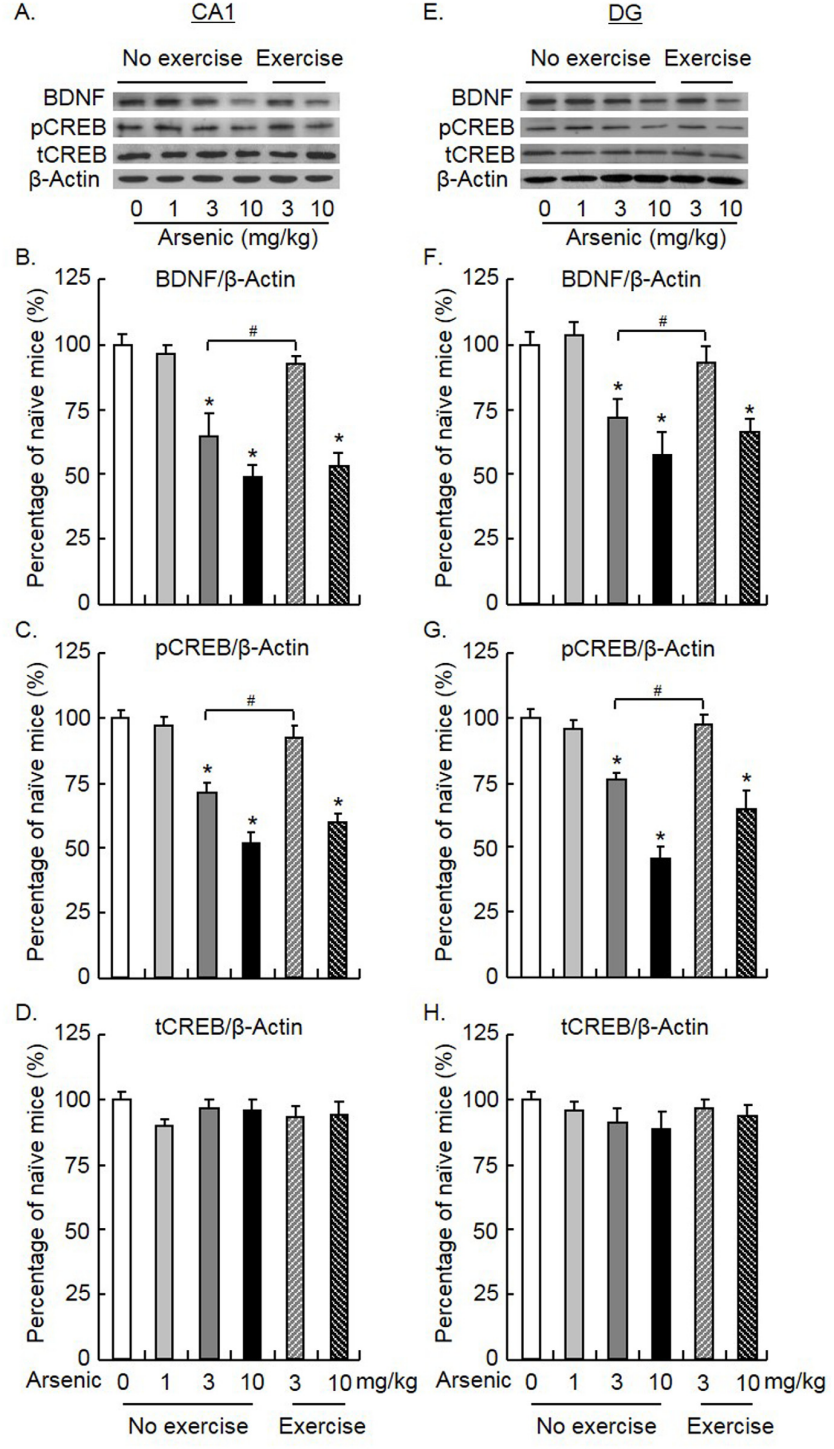
Exercise prevented the decrease in hippocampal BDNF induced by low doses of arsenic exposure. Mice were administered arsenic and underwent swimming exercise daily for 12 weeks and then were decapitated 24 h later. **(A, E)** Representative Western blotting analyses of proteins in the CA1 and dentate gyrus (DG) areas of the dorsal hippocampus. **(B, F)** Exposure to arsenic at dosages of 3 and 10 mg/ kg decreased BDNF expression in CA1 and DG (both *p* < 0.05). Exercise prevented the decrease in BDNF expression in both CA1 and DG induced by arsenic at a dose of 3 mg/ kg (both *p* < 0.05), but not 10 mg/ kg (both *p* > 0.05). **(C, G)** The levels of phosphorylated CREB in CA1 and DG were reduced by arsenic at dosages of 3 and 10 mg/ kg (both *p* < 0.05). The decrease in pCREB expression in CA1 and DG induced by arsenic at dosages of 3 mg/ kg (both *p* < 0.05), but not 10 mg/ kg (both *p* > 0.05), was prevented by exercise. **(D, H)** Arsenic at any dosages had no effect on the levels of total CREB in CA1 and DG (both *p* > 0.05). The data were expressed as the mean ± SEM (n = 8 per group). *p < 0.05, compared with the group of mice that underwent no exercise and were treated with 0 mg/ kg arsenic; ^#^ *p* < 0.05, compared with the group of mice that underwent no exercise and were treated with 3 mg/ kg arsenic.

A recent study demonstrated that arsenic reduced BDNF expression in human neuroblastoma SH-SY5Y cells [13]. Hippocampal BDNF is critical for object recognition memory, which is mediated by CREB [24]. In this experiment, we tested whether arsenic exposure and swimming exercise could alter hippocampal BDNF expression and CREB activity. Six groups of mice were included (n = 8 per group): mice treated with 0, 1, 3, and 10 mg/ kg arsenic that underwent no exercise; mice treated with 3 and 10 mg/ kg arsenic that underwent exercise. For BDNF expression in the CA1 and DG, one-way ANOVA showed significant effects (CA1: *F*_5, 47_ = 19.32, *p* < 0.001; DG: *F*_5, 47_ = 10.94, *p* < 0.001). Post hoc analysis showed that 3 and 10 mg/ kg arsenic exposure reduced BDNF expression (all *p* < 0.001). Exercise prevented the decrease in BDNF induced by 3 mg/ kg (both *p* < 0.001), but not 10 mg/ kg (both *p* > 0.05), or arsenic exposure (Fig 4B and 4F). For phosphorylated CREB (pCREB) in the CA1 and DG, one-way ANOVA also showed significant effects (CA1: *F*_5, 47_ = 31.47, *p* < 0.001; DG: *F*_5, 47_ = 25.04, *p* < 0.001). Post hoc analysis showed that the expression of pCREB in both the CA1 and DG was reduced by 3 and 10 mg/ kg arsenic exposure (all *p* < 0.01). Moreover, a decrease in pCREB induced by 3 mg/ kg (*p* < 0.05), but not 10 mg/ kg (*p* > 0.05), of arsenic exposure was blocked by exercise (Fig 4C and 4D). For total CREB (tCREB) in the CA1 and DG, one-way ANOVA also showed no effect (CA1: *F*_5, 47_ = 0.77, *p* > 0.05; DG: *F*_5, 47_ = 0.85, *p* > 0.05). Taken together, these results indicated that chronic arsenic exposure reduced hippocampal BDNF/pCREB expression. Exercise prevented the decrease in BDNF/ pCREB induced by low dosages of arsenic, which suggested that arsenic exposure might alter LTM performance via hippocampal BDNF/ pCREB.

## Discussion

In the present study, we investigated the effect of arsenic exposure on object recognition memory, and tested whether this effect could be affected by swimming exercise. We found that chronic administration of 3 mg/ kg and 10 mg/ kg arsenic reduced body weight gain and increased arsenic content in the brain. Object recognition LTM was disrupted by 3 mg/ kg and 10 mg/ kg arsenic exposure. Swimming exercise prevented memory impairment induced by arsenic at dosages of 3 mg/ kg, but not 10 mg/ kg. The decrease in BDNF and pCREB in CA1 and DG induced by 3 mg/ kg, but not 10 mg/ kg, of arsenic was blocked by exercise.

Previous studies have demonstrated that arsenic exposure decreased body weight, which is consistent with the present findings that higher doses of arsenic exposure induced more severe weight loss and higher arsenic content in the brain. The decrease in body weight might be induced by the reduction in the repair and synthetic activities of various cells, but not a decrease in food intake [10,25–27]. However, the present results also indicated that intragastric administration of arsenic in mice is a suitable model to study the toxic effects of chronic arsenic administration.

The toxic effects of arsenic on memory performance in rodents have been previously reported. The performance of rats in the Morris water maze and Y maze tasks were impaired by low-dose sodium arsenic exposure [8]. Learning and memory in the Morris water maze was disrupted in rats that were exposed to arsenic, and this effect was not promoted by co-administration of fluoride [12]. Rats treated with arsenic showed a significantly decreased learning activity of passive avoidance response [28]. Consistent with these results, we found that object recognition LTM was disrupted by arsenic exposure. Although all dosages of arsenic in the present study decreased body weight gain and increased the arsenic content in the brain, mice exposed to 1 mg/ kg arsenic showed no memory impairment, which indicated that cognitive function was resistant to arsenic toxicity to some extent. The highest dosage of arsenic exposure showed a decreasing trend of total exploring time towards two objects during the LTM tests, indicating that arsenic exposure could also affect locomotor activity [29]. It was demonstrated that motor activity was increased by arsenic trioxide at 3 mg/kg and decreased by arsenic trioxide at 10 mg/kg for two weeks [29]. A recent study investigated the effect of arsenic exposure for 4 months on the locomotor activity of mice [30]. They found that male mice exposed to 0.5 mg As/L in drinking water showed hyperactivity, whereas male mice exposed to 50 mg As/L presented hypoactivity and male mice exposed to 5 mg As/L did not show a significant difference with control groups of mice, indicating that the effect of arsenic on locomotion was dose-dependent. In the present study, we found no difference in the total exploration time among mice exposed to 0, 1, and 3 mg/kg arsenic, indicating that the memory impairment induced by 3 mg/kg arsenic was not due to locomotor alteration in mice.

Numerous studies have investigated the protective effects of several agents on arsenic-induced damage. Oral treatment with tephrosia purpurea extract protected rats from arsenic-induced nephrotoxicity [31]. In addition, Juglans nigra showed hepatoprotective and anticlastogenic effects induced by arsenite [32]. Pre-treatment with the extract of V. amygdalina suppressed chromosomal aberration induced by sodium arsenite in rats [33]. In studies of protection against arsenic-induced oxidative damage, it was suggested that dietary flaxseed oil, green tea, melatonin, N-acetyl cysteine, selenium, psidium guajava, corchorus olitorius, and melatonin supplementation could be protective agents against arsenic-induced cellular oxidative stress [34–41]. Moreover, recent studies have demonstrated that curcumin showed neuroprotective efficacy in attenuating arsenic neurotoxicity [42,43]. Furthermore, curcumin promoted learning and memory performance in rats treated with arsenic [28]. Another effective tool, which has been shown to improve cognitive performance, is physical exercise [14,44–46]. In the present study, we found that swimming exercise [47,48] prevented memory impairment induced by 3 mg/ kg arsenic, indicating that swimming exercise could be a non-pharmacological tool used to reduce arsenic neurotoxicity and improve cognitive function. It should be noticed that, it is possible that the forced swimming, as a kind of mild stress, would exacerbate the poisoning of arsenic when the concentration of arsenic is very high.

Furthermore, several studies indicated that exercise promotes cognition and memory. Treadmill exercise improved spatial learning memory [49,50]. A study investigating the effect of exercise on different stages of memory revealed that treadmill running improved the consolidation of passive avoidance memory but had no effect on memory retrieval [51]. Moreover, physical exercise improved cognitive performance in psychogeriatric patients [14]. In addition, swimming alone improved elevated plus maze-associated memory and prevented emotional memory impairment induced by the infusion of scopolamine into CA1 [47]. Because the effect of exercise on cognition improvement requires a long period of time, it was suggested that exercise contributed to memory improvement by promoting neurogenesis in the hippocampus [45,46]. Considering the limitations in the NOR task, for example the exploratory activity will vary when using different open-field arenas or food deprivation [52], we cannot generalize our findings to other kinds of exercise, e.g. treadmill exercise, which requires further investigation. Moreover, we cannot exclude other possible effect but not exercise itself in the swimming training, e.g. “environmental enrichment” effect.

The mechanism underlying cognitive impairment caused by chronic arsenic exposure in rodents has only been recently investigated. One of the most widely studied brain areas affected by arsenic is the hippocampus, a critical brain area that participates in memory performance. First, arsenic exposure induced structural alterations in the brain. An ultra-structural study showed that chronic exposure to drinking water containing arsenic induced swollen mitochondrion and expanded the rough endoplasmic reticulum in rat hippocampal neurons [10]. In addition, abnormal structural alterations in the myelin sheaths of nerve fibers and a reduction in the terminals of mossy fibers in the hippocampus were also observed [8]. Second, arsenic exposure induced alterations in several molecules in the hippocampus. Among them, BDNF, a crucial factor involved in learning and memory, interacts with these molecules to regulate gene transcription and protein synthesis in the formation of LTM [53,54]. In the present study, we demonstrated that BDNF/phosphorylated CREB (pCREB) in the CA1 and DG of the hippocampus was reduced in mice exposed to arsenic.

Importantly, a decrease in BDNF/ pCREB was prevented by swimming exercise. BDNF has been reported to be an important mediator in cognition enhancement. BDNF, interacting with other plasticity related proteins, mediates exercise-induced hippocampal plasticity [55]. Physical exercise reversed high-fat diet-induced hippocampal-dependent memory impairment and increased BDNF expression in CA3 of the hippocampus [22]. Inhibition of hippocampal BDNF blocked the effect of exercise on performance in the Morris water maze and altered CREB and synapsin I levels, which are important for synaptic plasticity [19]. BDNF may also interact with AMP-activated protein kinase, insulin-like growth factor I, and ghrelin to regulate exercise-induced synaptic plasticity and cognitive function [56,57]. Voluntary exercise significantly promoted short-term and long-term spatial memory, and increased BDNF expression in the hippocampus [58]. Improvement of hippocampus-related cognitive functions by voluntary resistance wheel running was also associated with hippocampal BDNF signaling [59]. Swimming exercise, used in the present study, also showed an enhanced effect on spatial memory, which may be mediated by BDNF [21,60]. Together with the behavioral alterations induced by arsenic, the present results suggest that hippocampal BDNF/CREB may play a critical role in learning and memory in mice exposed to arsenic. However, the precise mechanism how is BDNF/CREB pathway involved in arsenic neurotoxicity requires further studies. Furthermore, The effect of swimming exercise may reduce arsenic poisoning in two ways. First, swimming may directly reduce the level of arsenic via accelerating arsenic elimination. Second, swimming may reduce the effect of arsenic in the brain through activating some protecting process. Further study is also required to test the persistence of the effect of swimming exercise on arsenic poisoning, which is very important in clinical.

In summary, arsenic exposure impaired long-term object recognition memory, which was partially prevented by swimming exercise in mice. Hippocampal BDNF/CREB expression was associated with memory ability regulated by arsenic and exercise. The exercise paradigm in mice used in this study can be readily transferred to clinical treatment, thus understanding the mechanism underlying arsenic-induced memory impairment would provide therapeutic insight towards a treatment for arsenic neurotoxicity.

## Supporting Information

S1 ARRIVE Checklist NC3Rs ARRIVE Guidelines Checklist. (PDF)
